# To what degree is palliative care integrated in guidelines and pathways for adult cancer patients in Europe: a systematic literature review

**DOI:** 10.1186/s12904-016-0100-0

**Published:** 2016-03-03

**Authors:** Karen Van Beek, Naouma Siouta, Nancy Preston, Jeroen Hasselaar, Sean Hughes, Sheila Payne, Lukas Radbruch, Carlos Centeno, Agnes Csikos, Eduardo Garralda, Marlieke van der Eerden, Farina Hodiamont, Ildiko Radvanyi, Johan Menten

**Affiliations:** Department of Radiation-Oncology and Palliative Medicine, University Hospital Gasthuisberg, Leuven, Belgium; International Observatory on End of Life Care, Division of Health Research Lancaster University, Lancaster, United Kingdom; Department of Anesthesiology, Pain and Palliative Medicine, Radboud University Nijmegen Medical Centre, Nijmegen, The Netherlands; Department of Palliative Medicine, University Hospital Bonn, Bonn, Germany; Department of Palliative Medicine, University of Navarra Hospital, Pamplona Navarra, Spain; Faculty of Medicine, Institute of Family Medicine, University of Pécs Medical School, Pécs, Hungary

**Keywords:** Delivery of Health Care, Integrated, Palliative care, Medical oncology, Systematic review, Guidelines, Pathways

## Abstract

**Background:**

Palliative Care (PC) aims to improve the quality of life for patients with cancer and their families and its benefits have been demonstrated by several studies. The objective of this systematic review is to assess the integration of PC in the content of guidelines/pathways of adult cancer patients in Europe.

**Methods:**

We included studies of adult patients with cancer published from 01/01/1995 and 31/12/2013 in Europe in six languages. We searched nine electronic databases, hand-searched six journals and also performed citation tracking. Studies were ranked using Emanuel’s Integrated Palliative Care (IPC) criteria, a tool containing 11 domains to assess PC content in guidelines. Two reviewers screened the results and narrative synthesis has been employed.

**Results:**

We identified a total of 28,277 potentially relevant articles from which 637 were eligible for full-text screening. The final review included 60 guidelines and 14 pathways. Eighty percent (80 %) of the guidelines/pathways emphasize a holistic approach and 66 % focus on PC interventions aimed at reducing suffering. Fifty seven percent (57 %) did not discuss referral criteria for PC. Of all studies, five fulfilled at least 10/11 IPC criteria. Differences existed with regard to the referral criteria for bereavement care and the continuous adjustment of goals of care.

**Conclusion:**

Overall, most of the identified guidelines/pathways highlighted the importance of the holistic approach of IPC. The studies that were found to fulfil at least 10/11 Emanuel’s IPC criteria could serve as benchmarks of IPC.

## Background

According to EUROSTAT [1], cancer constitutes the second most common cause of death in the European Union, which amounts for 29 and 23 % of deaths for men and women, respectively. Moreover, these numbers are expected to increase as a result of the ageing of the population.

Palliative Care (PC) amounts to optimizing the quality of life for patients and their families facing problems associated with cancer, and other life-threatening disease more generally. In this framework, focus is placed on i) the alleviation of symptoms, ii) the up-to-date communication of treatment goals and iii) the support for both patients and their families throughout the course of the illness trajectory. Importantly, the effectiveness of palliative care on the improvement of the quality of life of patients with advanced cancer has been corroborated by an ever-growing bulk of research evidence [2–6].

Several health-care authorities and medical associations including the World Health Organization (WHO), the European Society of Medical Oncology (ESMO), the American Society of Clinical Oncology (ASCO), The National Comprehensive Cancer Network (NCCN), The European Association for Palliative Care (EAPC) and Institute for Clinical Systems Improvement (ICSI) recommend the (early) integration of PC in the illness trajectory [2, 7–13]. However, available studies report that, at the end of life, patients with cancer usually receive suboptimal care [14], i.e. integration of PC remains limited. A critical examination of the status quo in Europe reveals that current European health-care delivery for patients with cancer is suboptimal both from the quality-of-care and from the financial perspective. In turn, this sub-optimality is associated with a multitude of adverse effects, a non-exhausting list of which includes: i) patients unable to die at their place of preference [15–17], ii) large discrepancies in treatment trajectories and (non)treatment strategies in those with advanced cancer, indicating limited consensus on optimal treatment pathways [18, 19] and iii) considerable risks for overburdening of informal caregivers, translating into an imbalance in care networks surrounding the patient [20–23].

Central to the successful development and implementation of integrated PC strategies are the concepts of guidelines and pathways. Guidelines are systematically developed statements to assist practitioners and patient decisions about appropriate health care for specific clinical circumstances. They can be national, international or local. As such, they are often used as a means to reduce variations in treatments within health-care systems, to develop hospital-tailored protocols, to educate students, to assist insurers etc., [24–26]. On the other hand, a care pathway is defined as a complex intervention for the mutual decision making and organisation of care processes for a well-defined group of patients during a well-defined period. A pathway may use guidelines to provide clinical care.

The objective of the present study is to identify existing guidelines and pathways of integrated PC for people with cancer in the European Union and evaluate their completeness of content regarding their level of PC integration by conducting a systematic review of the available literature.

## Methods

Despite the fact that there is a growing awareness of integrated palliative care (IPC) a unanimously agreed definition for it does not exist. For the scopes of this study, the InSup-C experts developed a consensus based definition that reads:

“Integrated palliative care involves bringing together administrative, organisational, clinical and service aspects in order to realise continuity of care between all actors involved in the care network of patients receiving palliative care. It aims to achieve quality of life and a well-supported dying process for the patient and the family in collaboration with all the care givers (paid and unpaid)”.

### Search strategy

The following databases were searched electronically: The Cochrane Central Register of Controlled Trials (CENTRAL), PubMed, EMBASE, CINAHL, AMED, British Nursing index (BNI), Web of Science, National Guidelines Clearinghouse and NHS Evidence. The search in the databases was performed using judiciously chosen keywords and search terms as well as their permutations/combinations. The basic search terms and keywords that were used in the electronic databases are presented in Table 1.

**Table 1:** Search terms used for the database search

(hospices OR supportive care OR supportive care* OR end of life care* OR palliative* OR palliative care [MeSH Terms] OR hospice* OR terminal care* OR coordinated care* OR integrated care* OR transmural care* OR progressive patient care*) AND (“end stage disease” OR end stage disease* OR dying OR death [MeSH Terms] OR Chronic disease [MeSH Terms] OR Chronic disease* OR terminally ill* OR terminally ill [MeSH Terms] OR cancer) AND (care pathway* OR care pathway OR pathway* OR patient transfer* OR patient transfer OR patient care team* OR managed care program* OR continuity of patient care OR patient care management OR patient care plan* OR patient care planning OR illness trajectory OR “advance care planning” OR advance care planning OR delivery of health care OR models of care OR model of care OR model organizational OR models organizational OR organizational model* OR guideline*) NOT ((birth) OR child) OR pediatrics)) NOT ((animals[mh] NOT humans[mh])) Filters: Publication date from 1995/01/01 to 2013/12/31	

Additionally, the following journals were hand-searched: BMJ Supportive & Palliative care, European Journal of Palliative Care, Journal of Pain and Symptom Management, Palliative Medicine, Medicina Paliativa and the references from the included guidelines/pathways.

The grey literature search consisted of two parts. For the first part, named individuals within national scientific medical organizations, were contacted with the aim of gathering information on guidelines and pathways. Examples of professional organizations included national bodies for oncology and palliative care development. The second part consisted of a grey literature search involving an electronic search in Google. For this search, the search strategy was translated in the languages of the authors participating in this study.

### Selection criteria

As mentioned in the introduction, this study is performed in the context of the InSup-C research project. For this reason, in compliance with the project’s objectives this review is geographically limited in Europe. Further, the following inclusion criteria were used:

1. Guidelines and pathways for adult patients.
2. Guidelines and pathways for cancer (latest possible versions).
3. European guidelines and pathways.
4. Guidelines and pathways published from 01-01-1995 to 31-12-2013 (with the start date based on the publication of the Calman-Hine report which constitutes the first national cancer plan in Europe [27]).
5. Languages: English, French, German, Dutch, Hungarian and Spanish (the languages of the authors)

To distinguish between studies focusing on PC from those focusing on IPC, a sixth eligibility criterion is needed. In the present study, we measure the completeness of the IPC content of the guidelines/pathways via a tool based on Emanuel’s IPC criteria. This is a template designed by the American Hospice Foundation Guidelines Committee to provide a practical approach for guideline writers and others to integrate PC into disease management and care services whenever it is relevant [28]. These criteria are described in Table 2. For the needs of the present study, and following a consensus in the InSup-C consortium, a guideline/pathway is considered to focus on IPC if it fulfils at least two out of the 11 criteria. Therefore, guidelines/pathways that fulfilled at most one criterion were labelled as non-eligible.

**Table 2:** Integrated Palliative Care (IPC) Criteria

1. Discussion of illness limitations and prognosis. 2. Recommendations for conducting a whole patient assessment including the patient’s physical, social, psychological, and spiritual issues, their family and community setting. 3. Recommendations for when to make these assessments (e.g. At baseline and periodically thereafter). 4. Recommendations on when palliative care should be integrated. 5. Assessment of the patient’s goals for care. 6. Continuous goal adjustment as the illness and the person’s disease progresses. 7. Palliative care interventions to reduce suffering as needed. 8. Advance care planning. 9. Recommendation of involving a palliative care team (interdisciplinary team, palliative care consultation or other palliative care services). 10. Recommendations on care during the last hours of living. 11. Recommendations on grief and bereavement care.	

It is important to note that, to the best of our knowledge, Emanuel’s criteria constitute the only tool that evaluates the content of the guidelines/pathways regarding the level of PC integration. Consequently, even though this tool has not been empirically validated, it has been chosen as the best choice.

### Selection procedure

In the first phase, the first two authors (KVB and NS) screened all the English search results based on their titles and their abstract. Non English texts were screened and translated by two native speaker researchers. Then the full texts of the guidelines/pathways selected by both authors were sourced and they were reviewed based on the aforementioned inclusion/exclusion criteria. Discrepancies were resolved by consensus or they were extensively discussed in consecutive project meetings.

### Data extraction

Data were extracted from guidelines/pathways meeting the inclusion criteria using a data extraction form. This form was based on the extraction form described in Hawker et al. [29], but it was adjusted accordingly to the purposes of our study after consensus in the project meetings. For each included paper, data extraction was carried out by the two first authors independently for the English results and by two native speaker researchers for the non-English ones. Upon the completion of the process, the two reviewers cross-checked their results and reached consensus in case of discrepancies.

### Evidence quality assessment

In order to assess the quality of the evidence of the guideline/pathways, we employed the following methodology. Guidelines/pathways that were based on both systematic reviews and consensus methods (e.g. nominal group techniques, Delphi rounds, expert consultations) or those developed by following the NICE protocol [30] were considered high quality evidence; guidelines/pathways based on systematic review only or based on other types of well referenced evidence were considered medium quality whereas those based on consensus methods only were considered low quality. Finally, guidelines/pathways whose basis was unclear (e.g. apparently evidence based but failing to clarify how this was obtained) were considered of very low quality. This quality assessment guide was agreed upon during consensus between the authors.

### Data synthesis

The included studies are characterized by a substantial heterogeneity. For this reason, a narrative synthesis has been considered appropriate and results have tabulated in easily accessible tables (Tables 3 and 4).

**Table 3:** Characteristics of included Cancer Guidelines

Cancer guidelines			
Title/Country/Year	Setting	Emanuel’s criteria (EMC)	Quality of evidence
Interdisciplinary guideline of quality for early detection, diagnosis and treatment of different stages of prostate cancer/Germany/2011. [31]	inpatient/outpatient	7 EMC: Discussion of illness limitations and prognosis, Holistic assessments, Timing of PC introduction, Patient’s goals, Suffering reduction, ACP, Involvement of PC team.	High
Practice guideline for palliative care/Germany/2009. [32]	outpatient	9 EMC: Holistic assessments, Timing of PC introduction, Patient’s goals, Continuous goal adjustment, Suffering reduction, ACP, Involvement of PC team, Last hours of living care, Grief and bereavement care.	Low
Interdisciplinary guidelines for the diagnosis, treatment and follow-up of breast cancer/Germany/2012. [33]	inpatient/outpatient	9 EMC: Discussion of illness limitations and prognosis, Holistic assessments, Timing of holistic assessments, Timing of PC introduction, Patient’s goals, Continuous goal adjustment, Suffering reduction, Involvement of PC team, Last hours of living care.	High
Guideline: Prevention, Diagnosis, Therapy, and Follow-up of Lung Cancer. (Germany, 2010) [34]	inpatient/outpatient	9 EMC: Discussion of illness limitations and prognosis, Holistic assessments, Timing of PC introduction, Patient’s goals, Continuous goal adjustment, Suffering reduction, ACP, Involvement of PC team, Last hours of living care.	High
Malignant melanoma S3-guideline: Diagnosis, treatment and aftercare of melanoma. (Germany, 2013) [35]	inpatient/outpatient	7 EMC: Discussion of illness limitations and prognosis, Holistic assessments, Timing of holistic assessments, Timing of PC introduction, Suffering reduction, ACP, Involvement of PC team.	High
Cancer pain. (Germany, 2007) [36]	inpatient/outpatient	3 EMC: Holistic assessments, Timing of holistic assessments, Continuous goal adjustment.	High
Directive of the Federal Joint Committee on the Regulation of specialized outpatient palliative care. (Germany, 2007). [37]	outpatient	6 EMC: Holistic assessments, Patient’s goals, Suffering reduction, ACP, Involvement of PC team, Last hours of living care.	Very low
National Practice Guideline pancreatic cancer. (Belgium, 2009) [38]	inpatient	3 EMC: Holistic assessments, Timing of PC introduction, Involvement of PC team.	High
Small cell and non- small cell lung cancer: diagnosis, treatment and follow-up. (Belgium, 2013) [39]	inpatient	2 EMC: Patient’s goals, ACP.	High
National Practice Guideline of oesophageal and stomach cancer – UPDATE. (Belgium, 2012) [40]	inpatient	4 EMC: Holistic assessments, Timing of PC introduction, Suffering reduction, Involvement of PC team.	High
Palliative Care Unit: Standards and Recommendations. (Spain, 2009) [41]	inpatient/outpatient	8 EMC: Discussion of illness limitations and prognosis, Holistic assessments, Timing of PC introduction, Patient’s goals, Suffering reduction, ACP, Involvement of PC team, Grief and bereavement care.	High
Clinical Practice Guidelines on Palliative Care. (Spain, 2008) [42]	inpatient/outpatient	10 EMC: Discussion of illness limitations and prognosis, Holistic assessments, Timing of PC introduction, Patient’s goals, Continuous goal adjustment, Suffering reduction, ACP, Involvement of PC team, Last hours of living care, Grief and bereavement care.	High
Palliative Care Guideline. (Spain, no date available) [43]	inpatient/outpatient	3 EMC: Involvement of PC team, Last hours of living care, Grief and bereavement care.	Very low
Palliative Care Guideline in the Community of Madrid. (Spain, 2008) [44]	inpatient/outpatient	5 EMC: Discussion of illness limitations and prognosis, Holistic assessments, Timing of PC introduction, Suffering reduction, Last hours of living care.	Very low
Clinical recommendations guideline: colorectal cancer. (Spain, 2006). [45]		4 EMC: Holistic assessments, Timing of PC introduction, Continuous goal adjustment, Grief and bereavement care.	High
Guideline Care in the dying phase. (The Netherlands, 2010) [46]	inpatient/outpatient	8 EMC: Discussion of illness limitations and prognosis, Holistic assessments, Timing of PC introduction, Patient’s goals, Suffering reduction, ACP, Last hours of living care, Grief and bereavement care.	Low
Guideline Leptomeningeal metastases. (The Netherlands, 2010) [47]	inpatient/outpatient	4 EMC: Discussion of illness limitations and prognosis, Holistic assessments, Timing of PC introduction, Suffering reduction.	High
Guideline Oncologic Rehabilitation. (The Netherlands, 2011) [48]	inpatient/outpatient	6 EMC: Holistic assessments, Timing of holistic assessments, Timing of PC introduction, Patient’s goals, Continuous goal adjustment, Suffering reduction.	Medium
Guideline NSCLC. (The Netherlands, 2011). [49]	inpatient/outpatient	5 EMC: Discussion of illness limitations and prognosis, Holistic assessments, Timing of holistic assessments, Patient’s goals, Suffering reduction.	High
Guideline Melanoma. (The Netherlands, 2013) [50]	inpatient/outpatient	3 EMC: Discussion of illness limitations and prognosis, Timing of PC introduction, Suffering reduction.	High
Guideline Oesophagus carcinoma. (The Netherlands, 2010) [51]	inpatient/outpatient	8 EMC: Discussion of illness limitations and prognosis, Holistic assessments, Timing of holistic assessments, Timing of PC introduction, Patient’s goals, Continuous goal adjustment, Suffering reduction, ACP.	High
Guideline Pancreas carcinoma. (The Netherlands, 2011) [52]	inpatient/outpatient	3 EMC: Discussion of illness limitations and prognosis, Patient’s goals, Suffering reduction.	High
Guideline cervix carcinoma. (The Netherlands, 2012) [53]	inpatient/outpatient	4 EMC: Discussion of illness limitations and prognosis, Holistic assessments, Suffering reduction, ACP, Involvement of PC team.	High
Guideline Endometrial carcinoma. (The Netherlands, 2011) [54]	inpatient/outpatient	2 EMC: Discussion of illness limitations and prognosis, Involvement of PC team.	High
Guideline Sarcoma carcino-sarcoma uterus. (The Netherlands, 2010) [55]	inpatient/outpatient	2 EMC: Holistic assessments, Involvement of PC team.	Low
Guideline Hypo-pharynx carcinoma. (The Netherlands, 2010) [56]	inpatient/outpatient	5 EMC: Discussion of illness limitations and prognosis, Holistic assessments, Timing of holistic assessments, Timing of PC introduction, Suffering reduction.	High
Guideline Larynx carcinoma. (The Netherlands, 2010) [57]	inpatient/outpatient	5 EMC: Discussion of illness limitations and prognosis, Holistic assessments, Timing of holistic assessments, Timing of PC introduction, Suffering reduction.	High
Guideline Mouth and oropharynx carcinoma. (The Netherlands, 2004) [58]	inpatient/outpatient	5 EMC: Discussion of illness limitations and prognosis, Holistic assessments, Timing of holistic assessments, Timing of PC introduction, Suffering reduction.	High
Guideline Breast cancer. (The Netherlands, no date available) [59]	inpatient/outpatient	4 EMC: Discussion of illness limitations and prognosis, Holistic assessments, Timing of holistic assessments, Suffering reduction.	High
Guideline Prostate carcinoma. (The Netherlands, 2007) [60]	inpatient/outpatient	3 EMC: Discussion of illness limitations and prognosis, Holistic assessments, Patient’s goals.	High
Professional Guideline (Directive) of the Hungarian Public Healthcare. (Hungary, 2013) [61]	inpatient	10 EMC: Discussion of illness limitations and prognosis, Holistic assessments, Timing of holistic assessments, Patient’s goals, Continuous goal adjustment, Suffering reduction, ACP, Involvement of PC team, Last hours of living care, Grief and bereavement care.	Medium
Recommendations for the development of an integrative and complex palliative care in Hungary. (Hungary, 2013) [62]	inpatient/outpatient	8 EMC: Discussion of illness limitations and prognosis, Timing of holistic assessments, Timing of PC introduction, Patient’s goals, Continuous goal adjustment, Suffering reduction, ACP, Grief and bereavement care.	High
Guidance on Cancer Services: Improving Supportive and Palliative Care for Adults with Cancer. (UK, 2004) [63]	inpatient/outpatient	9 EMC: Discussion of illness limitations and prognosis, Holistic assessments, Timing of holistic assessments, Patient’s goals, Suffering reduction, ACP, Involvement of PC team, Last hours of living care, Grief and bereavement care.	High
Making good care better: National practice statements for general palliative care in adult care homes in Scotland. (UK-Scotland, 2006) [64]	outpatient	8 EMC: Holistic assessments, Patient’s goals, Continuous goal adjustment, Suffering reduction, ACP, Involvement of PC team, Last hours of living care, Grief and bereavement care.	Low
The diagnosis and treatment of lung Cancer-updated. (UK, 2011) [65]	inpatient	4 EMC: Discussion of illness limitations and prognosis, Holistic assessments, Timing of holistic assessments, Suffering reduction.	High
Head and Neck Cancer: Multidisciplinary Management Guidelines. (UK, 2011) [66]	inpatient/outpatient	7 EMC: Discussion of illness limitations and prognosis, Holistic assessments, Patient’s goals, Continuous goal adjustment, Suffering reduction, Involvement of PC team, Last hours of living care.	High
Guidelines for supportive care in multiple myeloma 2011. (UK, 2011) [67]	inpatient	4 EMC: Holistic assessments, Timing of holistic assessments, Suffering reduction, ACP, Last hours of living care.	High
The NICE Guidance on Supportive and Palliative Care Implications for Oncology Teams. (UK, 2004) [68]	inpatient	5 EMC: Holistic assessments, Patient’s goals, Suffering reduction, Involvement of PC team, Grief and bereavement care.	High
Metastatic malignant disease of unknown primary origin. Diagnosis and management of metastatic malignant disease of unknown primary origin. (UK, 2010) [69]	inpatient	3 EMC: Discussion of illness limitations and prognosis, Holistic assessments, Continuous goal adjustment.	High
Palliative and End of Life Care Indicators. (UK-Scotland, 2013) [70]	inpatient/outpatient	3 EMC: Discussion of illness limitations and prognosis, Patient’s goals, ACP.	Low
Core competencies in palliative care: an EAPC White Paper on palliative care education. Parts 1 & 2. (UK, 2013) [71]	inpatient/outpatient	8 EMC: Discussion of illness limitations and prognosis, Holistic assessments, Timing of holistic assessments, Patient’s goals, Continuous goal adjustment, ACP, Involvement of PC team, Grief and bereavement care.	Low
Dying well at home: the case for integrated working: Guide 48. (UK, 2013) [72]	inpatient/outpatient	7 EMC: Holistic assessments, Patient’s goals, Suffering reduction, ACP, Involvement of PC team, Last hours of living care, Grief and bereavement care.	Low
RCGP commissioning guidance in end of life care : guidance for GPs, clinical commissioning group advisers. (UK, 2013) [73]	inpatient/outpatient	7 EMC: Discussion of illness limitations and prognosis, Holistic assessments, Patient’s goals, Continuous goal adjustment, Involvement of PC team, Last hours of living care, Grief and bereavement care.	Low
Optimising the role and value of the interdisciplinary team: providing person centred end of life care. (UK, 2013) [74]	inpatient/outpatient	9 EMC: Discussion of illness limitations and prognosis, Holistic assessments, Timing of holistic assessments, Patient’s goals, Continuous goal adjustment, ACP, Involvement of PC team, Last hours of living care, Grief and bereavement care.	Low
Strategy for adult palliative and end of life care services. (UK, 2013) [75]	inpatient/outpatient	8 EMC: Discussion of illness limitations and prognosis, Holistic assessments, Patient’s goals, Continuous goal adjustment, Suffering reduction, Involvement of PC team, Last hours of living care, Grief and bereavement care.	Low
End of Life Care Strategy: Fourth Annual Report. (UK, 2012) [76]	inpatient/outpatient	3 EMC: ACP, Last hours of living care, Grief and bereavement care.	High
Matters of life and death: helping people to live well until they die. General practice guidance for implementing the RCGP/RCN end of life care patient charter. (UK, 2012) [77]	inpatient/outpatient	11 EMC: Discussion of illness limitations and prognosis, Holistic assessments, Timing of holistic assessments, Timing of PC introduction, Patient’s goals, Continuous goal adjustment, Suffering reduction, ACP, Involvement of PC team, Last hours of living care, Grief and bereavement care.	High
End of life care for adults in the Emergency Department. (UK, 2012) [78]	inpatient	4 EMC: Discussion of illness limitations and prognosis, Timing of PC introduction, ACP, Involvement of PC team.	High
CMG42 End of life care for adults. (UK, 2011) [79]	inpatient/outpatient	11 EMC: Discussion of illness limitations and prognosis, Holistic assessments, Timing of holistic assessments, Timing of PC introduction, Patient’s goals, Continuous goal adjustment, Suffering reduction, ACP, Involvement of PC team, Last hours of living care, Grief and bereavement care.	High
Commissioning guidance for specialist palliative care : helping to deliver commissioning objectives. (UK, 2012) [80]	inpatient/outpatient	6 EMC: Discussion of illness limitations and prognosis, Holistic assessments, Timing of PC introduction, Patient’s goals, ACP, Involvement of PC team.	Low
Commissioning person centred end of life care. (UK, 2012) [81]	inpatient/outpatient	2 EMC: ACP, Involvement of PC team.	Low
Quality standard for end of life care for adults. (UK, 2011) [82]	inpatient/outpatient	8 EMC: Holistic assessments, Timing of holistic assessments, Patient’s goals, Continuous goal adjustment, ACP, Involvement of PC team, Last hours of living care, Grief and bereavement care.	High
Advanced breast cancer: Diagnosis and treatment. (UK, 2009) [83]	inpatient/outpatient	3 EMC: Holistic assessments, Timing of holistic assessments, Involvement of PC team.	High
Review of palliative care services in Scotland. (UK-Scotland, 2008) [84]	inpatient/outpatient	4 EMC: Holistic assessments, Patient’s goals, ACP, Involvement of PC team.	High
Living and Dying Well: A national action plan for palliative and end of life care in Scotland. (UK-Scotland, 2008) [85]	inpatient/outpatient	8 EMC: Discussion of illness limitations and prognosis, Holistic assessments, Timing of holistic assessments, Timing of PC introduction, Patient’s goals, Suffering reduction, ACP, Involvement of PC team.	Low
Metastatic spinal cord compression: Diagnosis and management of patients at risk of or with metastatic spinal cord compression. (UK, 2008) [86]	inpatient/outpatient	2 EMC: Holistic assessments, Involvement of PC team.	High
End of life care. (UK, 2008) [87]	inpatient/outpatient	5 EMC: Holistic assessments, Patient’s goals, ACP, Last hours of living care, Grief and bereavement care.	Medium
National Care Standards: Hospice Care. (UK, 2005) [88]	inpatient/outpatient	2 EMC: Last hours of living care, Grief and bereavement care.	Low
Clinical Standards: Specialist palliative care. (UK, 2002) [89]	inpatient	7 EMC: Discussion of illness limitations and prognosis, Holistic assessments, Patient’s goals, Continuous goal adjustment, ACP, Involvement of PC team, Grief and bereavement care.	Low
Improving outcomes in gynaecological cancer: The Manual. (UK, 1999) [90]	inpatient/outpatient	2 EMC: Holistic assessments, Suffering reduction.	High

**Table 4:** Characteristics of included Cancer Pathways

Cancer pathways			
Title/Country/Year	Setting	Emanuel’s criteria (EMC)	Quality of evidence
Palliative Medicine: Essays - Reports - Discussion Posts - Comments: Liverpool Care Pathway Practical assistance. (Germany, 2008) [91]	inpatient	7 EMC: Holistic assessments, Timing of PC introduction, Patient’s goals, Continuous goal adjustment, Suffering reduction, ACP, Last hours of living care.	Very low
Practice and opportunities of the Hungarian hospice care provided at home. (Hungary, 2013) [92]	Inpatient/outpatient	8 EMC: Discussion of illness limitations and prognosis, Holistic assessments, Timing of holistic assessments, Timing of PC introduction, Patient’s goals, Continuous goal adjustment, Suffering reduction, Last hours of living care.	Low
Palliative care pathway in General Practice. (Belgium, 2012) [93]	outpatient	11 EMC: Discussion of illness limitations and prognosis, Holistic assessments, Timing of holistic assessments, Timing of PC introduction, Patient’s goals, Continuous goal adjustment, Suffering reduction, ACP, Involvement of PC team, Last hours of living care, Grief and bereavement care.	High
Integrated Oncological Pathways: prostate carcinoma. (The Nederlands, 2010) [94]	inpatient/outpatient	5 EMC: Discussion of illness limitations and prognosis, Holistic assessments, Suffering reduction, Involvement of PC team, Last hours of living care.	Medium
Integrated Oncological Pathways: colon-rectum carcinoma. (The Nederlands, 2010) [94]	inpatient/outpatient	4 EMC: Timing of PC introduction, Suffering reduction, Involvement of PC team, Last hours of living care.	High
Flow chart glioblastoma. (The Nederlands, 2012) [96]	inpatient/outpatient	4 EMC: Timing of holistic assessments, Timing of PC introduction, Involvement of PC team, Last hours of living care.	Very Low
Manual for the management of patients in palliative care in outpatient ER. (Spain, 2011) [97]	outpatient	4 EMC: Discussion of illness limitations and prognosis, Timing of PC introduction, Suffering reduction, Last hours of living care.	Low
Home care program in primary care. (Spain, 2004) [98]	outpatient	6 EMC: Holistic assessments, Timing of PC introduction, Suffering reduction, Involvement of PC team, Last hours of living care, Grief and bereavement care.	Very low
Integrated care process of Palliative Care. (Spain, 2007) [99]	inpatient/outpatient	7 EMC: Holistic assessments, Timing of PC ntroduction, Suffering reduction, ACP, Involvement of PC team, Last hours of living care, Grief and bereavement care.	High
Palliative care in the oncologic patient. Documents for integrated care processes related to cancer management. (Spain, 2005) [100]	inpatient/outpatient	4 EMC: Holistic assessments, Suffering reduction, Involvement of PC team, Grief and bereavement care.	Very low
Care pathway for the last days of life. (UK-Wales, 2004) [101]	inpatient	8 EMC: Holistic assessments, Patient’s goals, Continuous goal adjustment, Suffering reduction, ACP, Involvement of PC team, Last hours of living care, Grief and bereavement care.	Very low
The route to success in end of life care: achieving quality for social work. (UK, 2012) [102]	inpatient/outpatient	8 EMC: Holistic assessments, Patient’s goals, Continuous goal adjustment, Suffering reduction, ACP, Involvement of PC team, Last hours of living care, Grief and bereavement care.	Low
Quality in melanoma care: a best practice pathway. (UK, 2012) [103]	inpatient/outpatient	4 EMC: Holistic assessments, Patient’s goals, ACP, Involvement of PC team.	High
Derbyshire End of Life Care Guidance: a pathway for supporting people in the last year of life. (UK, 2010) [104]	inpatient/outpatient	8 EMC: Holistic assessments, Timing of holistic assessments, Patient’s goals, Suffering reduction, ACP, Involvement of PC team, Last hours of living care, Grief and bereavement care.	Low

## Results

**Fig. 1:**
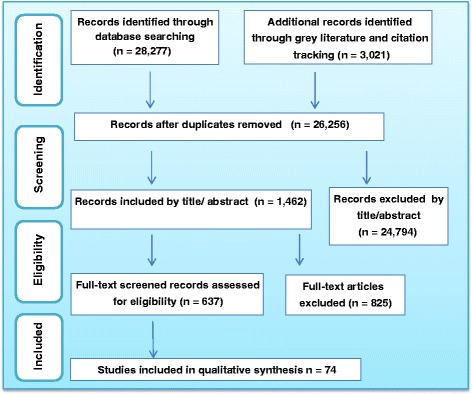
Flow diagram of study selection procedure

We identified a total of 28,277 potentially relevant documents/records, of which 24,794 were excluded based on their titles or abstracts. No additional articles were identified through consultation with experts and national health organizations. An additional 3021 relevant papers were identified from the hand-searched journals, citation tracking and the grey literature, of which 2905 were not eligible for full text screening. Full-text review was performed on the remaining 521 articles from the electronic database search and 116 articles from the other sources mentioned above. The final review included 74 papers of which 60 were guidelines [31–90] and 14 pathways [91–104]. The characteristics of these studies can be found in Tables 3 and 4. A flow diagram of the selection procedure and results (using the PRISMA tool [105]) is shown in Fig. 1.

Of the 60 guidelines included in the final review, 28 guidelines originated from the UK, seven from Germany, three from Belgium, five from Spain, 15 from the Netherlands and two from Hungary. Additionally, four pathways originated from the UK and Spain, three from the Netherlands and one from Belgium, Germany and Hungary. Twenty eight guidelines and five pathways can be considered as cancer specific (e.g. breast cancer guidelines, lung cancer guidelines, etc.). Four guidelines deal with general oncology, 11 guidelines and three pathways with the dying phase for patients with cancer and chronic disease, 16 guidelines and six pathways with PC in general and one guideline is about general health care. It is important to note here, that the guidelines/pathways focused on general PC and general health care are not irrelevant to the scopes of this systematic review because they are concerned with patients with cancer. In this respect, these guidelines/pathways fulfil the inclusion criterion on the type of the disease and as such they are included in the study. Further information on these guidelines and pathways is presented in Tables 3 and 4.

Looking at the results of the quality assessment of the evidence, we have to stress that the assessment employed in the present systematic review does not assess the quality of the implementation of the included guidelines/pathways. Rather, it provides a means of evaluating the principles upon which they have been developed. Very low quality guidelines/pathways corresponded to only 11 % of the total number. Guidelines/pathways with low quality were 27 %. Only a handful of studies (7 %) were categorised as medium quality. Finally, the majority of the included guidelines/pathways (55 %) were classified as of high quality evidence.

The assessment of the palliative care content of the guidelines/pathways via the IPC criteria revealed the following information. Nearly 80 % of the studies placed particular emphasis on the holistic approach, namely the assessment of the patient’s physical, psychological, social and spiritual issues, although only half of these recommendations (37 %) specified the exact timing on when these holistic assessments should take place. Of all guidelines/pathways, 47 % reported on the timing at which palliative care should be integrated. An additional 66 % of the guidelines/pathways focused on palliative care interventions aimed at reducing suffering. Interestingly, 75 % of the included guidelines/pathways referred to both an inpatient and an outpatient PC setting, as opposed to 16 and 8 % that referred solely to an inpatient and an outpatient PC setting, respectively. Moreover, 55 % of the guidelines/pathways explicitly put emphasis on discussion of illness limitations and prognosis and 55 % elaborated on the assessment of the patient’s goals for care.

Just less than half of the included guidelines/pathways paid attention to aspects of advance care planning (46 %) and grief and bereavement care (40 %). Furthermore recommendations for continuous goal adjustment (38 %), recommendations of care during the last hours of life (47 %) and recommendations for the involvement of a palliative care team (58 %), were mixed.

Guidelines and pathways that achieved a high score using the IPC criteria were judged to comprehensively address the core components of IPC. Two guidelines and one pathway scored 11/11 whereas two others scored 10/11. These five studies were also ranked as of high quality evidence which means that they were developed based on both consensus and literature review. Of interest is that these five studies are not specific oncology guidelines, but PC or End of Life Care guidance. The guidelines/pathways that scored 10 or 11/11 are presented and analysed in Table 5 and the strategies that they propose can be summarised as follows:

1. **Discussion of illness limitations and prognosis:** The guidelines/pathways agree that this can be realized through open and honest communication with patient and family, based on their needs and preferences, and enabling shared decision making. One pathway also suggests the employment of the surprise question or the Palliative Performance Scale can be used as triggers for initiating such discussions.
2. **Holistic assessment:** There is a unanimous consensus on the utilization of a combined physical, psychological, social and spiritual assessment.
3. **Timing of the holistic assessments:** Assessment should take place early in the disease trajectory. Further, it is recommended that holistic assessment should occur “at any time of day or night” for physical and psychological support and as long as possible for patient’s social participation. Also, its realization should vary depending on changes in the disease or on the appearance of new symptoms and based on application of e.g. a ‘distress thermometer’.
4. **Exact timing of introducing PC: Three strategies are identified: i)** the use of the surprise question, ii) the evaluation of the patient’s and the family’s needs, iii) illness stage – disease/cancer related prognostic indicators (e.g. like the indicators mentioned in the Gold Standards Framework).
5. **Patient’s goals assessments:** All the guidelines/pathways agree that this assessment should be based on the continuous communication between the patient and the PC specialists to identify patient goals.
6. **Continuous goal adjustment:** It is suggested that PC specialists regularly consult the patient and adjust goals accordingly.
7. **Suffering reduction:** The guidelines/pathways elaborate on the use of appropriate medication and strategies aimed in reducing both physical and psychological suffering.
8. **Advance care planning (ACP):** Decision making should be based on patient’s wishes and preferences. One pathway proposes the identification of the ACP via the use of three models (covenant model, contract model or DNR code).
9. **Involvement of PC team:** All of the guidelines/pathways strongly recommend the involvement or formation of a multidisciplinary PC team (consisting of physicians, nurses and other health professionals, psychologists, mental health counsellors, social workers, spiritual counsellors).
10. **Care during the last hours of living:** The following steps are recommended: identification of the dying phase, communication, support based on patients and family’s needs and wishes and symptom control.
11. **Grief and bereavement care:** The main proposed strategy involves the immediate and ongoing bereavement, emotional and spiritual support appropriate to the family’s needs and preferences.

**Table 5:** Top 5 ICP guidelines and pathways

IPC criteria	Clinical practice guidelines on palliative Care-Spain [42].	Guideline on the palliative care -Hungary [61].	Palliative care pathway in General Practice Country- Belgium [93].	General guidance RCGP/RCN- UK [77].	CMG42 End of life care for adults-UK [79].
Discussion of illness limitations and prognosis.	Communication should be based on patients’ and their family‘s needs rather than the expected survival time.	Information about the illness, decision making, and discussions about death should be based on the patient’s needs.	Description of surprise question and Palliative Performance Scale (PPS) to define prognosis or when to enter PC services.	Open and honest communication. Identification of triggers for discussion. Shared decision making.	Open communication and offering of information taking in account always the patient’s and family’s needs.
Holistic assessment.	Integral, frequent assessment in a multidisciplinary, individualized manner for: symptoms, pain, opioids use, spiritual needs, grief.	Whole patient assessment should include physical, psychosocial, and spiritual dimensions, according to the nature of the illness.	Recommendations on how to assess patients and how to deal with their physical, emotional, psychological, social and spiritual issues.	Holistic approach: physical, psychological, social, practical and emotional, religious and spiritual support.	Holistic approach: physical, psychological, social, emotional and spiritual support. Use of the Gold Standard Framework (GSF).
Timing for holistic assessments	Not included	assessment takes place at the first appointment. Further assessments depends on changes in the disease trajectory.	Whenever the patient is seen by the GP or nurse using the ‘distress thermometer’.	At any time of day or night for physical and psychological support and as long as possible for the social participation.	At any time of day or night for physical and psychological support and as long as possible for the social participation.
Timing for PC introduction	Interventions based on the patients &their family’s needs. PC services should be guaranteed when necessary.	Not included	Surprise Question: “Would you be surprised if your patient were to die in the next months, weeks, days?”.	Ask the Surprise Question “Would you be surprised if the patient were to die in the next months, weeks or days?”.	Timely access to generalist and specialist PC services on the basis of need and not diagnosis.
Patient’s goals assessments	Decision-making should be enhanced through the life goals and personal values.	Patient’s goals for care should be brought to light.	At the time of the holistic assessment, patients goals need to be assessed too.	Regular review of patients’ and carers’ needs and preferences.	Open conversations and clear expression of the end-of-life patients and their needs.
Continuous goal adjustment.	Needs on information and preferences of the patient should be assessed regularly.	Patients have the right for modifying the plan based according to their needs. Interventions should be adapted to patient’s goals.	Whenever there are changes in the disease trajectory patients goals need to be reassessed.	Discussions with patients and their carers about their future needs. This should be done as often as it feels that is needed.	Patients and carers should be offered holistic assessments in response to their changing needs and preferences.
Suffering reduction	Evaluation of the pain, instructions and involvement of patient in the use of analgesics and opioids depending of the pain stage and features.	Medical aspect of PC & applicable therapies, special treatments &interventions to reduce suffering (physical, psychosocial symptoms).	Based on an overview of needs of PC patients (study done by the Federal Knowledge Center), several caring goals to reduce suffering are given.	Should meet physical and psychological needs at any time of day or night, including access to medicines and equipment.	End of life patients should have their physical and psychological needs met at any time of day or night, including access to medicines and equipment.
Advance care planning (ACP).	Explore patient’s wishes and goals. Previous guidelines, wishes of the patient saved in his clinical records, legal and the nearest in charge relatives should be considered.	Patients have the right to information and autonomy/self-determination, refusal of treatments, & the process of making a living will	ACP can be done through 3 models (Covenant model, contract model or DNR code) and there are guidelines for urgent and non-urgent ACP.	Help the patients identify the choices that they may face, assist them to record their decisions and ensure that their wishes are fulfilled. Recognition of wishes for resuscitation, organ donation and place of death.	Increasing choice and personalization through ACP including advance decisions to refuse treatment and provision of resources that enable these choices.
Involvement of PC team.	Training of professionals to provide basic PC should be promoted; PC at any level should be provided preferably, by a multi-disciplinary team.	Implemented by a multi-disciplinary team; physicians, nurses, psychologists, mental health counsellors, social workers, clergymen.	If prognosis of <12 m, a multidisciplinary consultation will be organized between different health care professionals including PC.	Multidisciplinary generalist and specialist PC services should provide care over a 24 h period for people approaching the end of life.	Specialist multidisciplinary PC team should be responsive to emergency need and able to admit people approaching the end of life at any time
Recommendations on care during the last hours of living.	Recommendations include information, explanations, symptoms treatment, care continuity and holistic approach.	Symptoms and signs of death, reducing medication, nutrition, and fluid intake during the last hours of life.	Contains a separate section on how to identify the dying phase, communication, support, symptom control.	Identification of the dying phase (use of Patient Charter). Support for patient and carer. Use of the Liverpool Care Pathway.	Co-ordinated care across all relevant settings at any time, based on the person’s current medical condition, advance care planning and preferences.
Grief and bereavement care recommendations.	Identification of bereavement risk; interventions according to the nature of the grief, with professionals trained to deal with these issues.	Methods, aims and outcomes of bereavement counselling are described in the guideline.	Consultation after death, differentiation between depression, normal and complicated grief.	Timely verification and certification of death. Practical and emotional bereavement support for carer or family.	Immediate and ongoing, emotional, bereavement & spiritual support as appropriate to the needs and preferences of the carer/family.

The point at which PC should be introduced in the disease trajectory and which referral criteria should apply was addressed in less than half of the total of the included guidelines and pathways. This could be seen to have a significant impact on the benefits of PC if not introduced to patients in a timely manner. In view of this, it is surprising that 59 % (*n* = 43) of all the included guidelines/pathways did not discuss (adequately or at all) referral criteria.

The distribution of the guidelines/pathways that explicitly mentioned the referral criteria shows that 8 % recommended that the referral criterion should be for terminally ill people without specifying the exact timing. Further, 7 % reported that the referral criteria should be located in the last 6 months. Also, 4 % of the guidelines reported as a referral criterion the surprise question (“Would I be surprised if this patient died in the next year?”) whereas 8 % the reference of fulfilment of specific prognostic criteria mentioned in the Gold Standards Framework or Stadium IVB [106, 107]. The presence of metastatic disease was considered as a referral criterion by 15 % of all the studies whilst 13 % suggested the application of the guidelines as soon as the diagnosis of cancer has been made. Finally, one guideline recommended that the referral criteria should be determined upon a discussion with the patient and the family.

## Discussion

The results of the systematic review identified 74 guidelines/pathways fulfilling our inclusion criteria. Geographically, the majority of the included studies originated from the UK. Moreover, only five of them scored at least 10/11 of the IPC criteria.

Although there is significant improvement in cancer treatment, still half of all patients with cancer will eventually die of their disease and in one third this will happen within 6 months of diagnosis [108]. Currently, European and American oncology organisations such as ASCO, ESMO, and NCCN, recommend the early integration of PC for patients with cancer [2, 109]. These recommendations have been evidenced by studies that corroborate the positive relationship between early integration of PC and improvements in the quality of life of the patients, leading to better patient and caregiver outcomes, improvement of symptoms and patient satisfaction, with reduced caregiver burden, and reduced use of futile interventions [3, 12, 110–112]. Despite this evidence, cancer patients tend to be referred to PC late in their disease trajectory [12]. The implementation of PC integration is highly dependent on staff perception (misconception) of PC that often gets mistaken for terminal care [113].

The guidelines and pathways made a number of recommendations about how to introduce pc into oncology care. These included recommendations on how PC can be implemented earlier in oncology care are education of providers and public about the importance of PC and to coordinate PC efforts through strengthening affiliations and/or developing new partnerships [114]. One way to educate clinicians would be the integration of PC in disease specific guidelines.

According to our findings, most of the included guidelines/pathways recommend the need of a holistic PC approach e.g. a whole patient assessment. Additionally, it is also recognised that the reduction of suffering by implementing specific PC interventions e.g. use of opioids or analgesics, should be among the primary goals of integrated PC. This is in agreement with empirical evidence that illustrate the advantages of both the holistic approach and the focus on the reduction of suffering [115, 116].

A noteworthy outcome has been the general shortage in the guidelines of information on the specification on when PC should be initiated (PC referral criteria). Moreover, even among those guidelines/pathways that comment on the timing of referral, there is great variability e.g. surprise question, life expectancy less than 6 months, presence of metastatic disease, etc. We consider this important because clear referral criteria enable the initiation of the guideline/pathway. Consequently, this is correlated with the overall efficiency of a guideline/pathway. The notable absence of references to the referral criteria and the strong disagreement between those that mention them deserve further exploration.

More generally, there is a growing recognition that the PC should be considered around the time of diagnosis [117]. However, available research hints that that these recommendations alone are not sufficient for the practical determination of the referral criteria [118, 119]. With regard to cancer, a robust definition of the referral criteria is quite cumbersome and detailed and systematic methods need further development [113]. NCCN states that all patients should be screened for PC needs at their initial visit, at appropriate intervals and as clinically indicated [8]. For ASCO’s Panel’s expert consensus combined standard oncology care and palliative care should be considered early in the course of illness for any patient with metastatic cancer and/or high symptom burden [12]. In Germany, Gaertner et al. developed diagnosis specific guidelines for 19 malignancies to identify a disease- specific point in each disease trajectory to initiate palliative care [11]. In view of the above, we conclude that the establishment of PC referral criteria in guidelines for cancer patients remains an elusive and contentious topic which demands further attention.

Interestingly, guidelines/pathways give less attention to advance care planning, grief and bereavement support for the family members, and continuity of care. This outcome is surprising because the significance of these aspects has been highlighted by several studies [9, 120, 121]. Oncologists have to give their patients clear and consistent prognostic information as this will help facilitate discussions about patients’ end of life care preferences. Fears among oncologists that early PC consultation will frighten their patients are unfounded and the opposite has been proven [122]. Oncologists must ensure that advance care plans are in place as early as possible in the disease trajectory [8]. Bereavement support is gaining attention in the oncology field, with the NCCN guidelines being the first guideline that sees after-death care for the family as an essential part of the continuum of cancer care [8].

With the exception of the referral criteria, the holistic approach and the reduction of suffering, the compliance with Emmanuel’s IPC criteria of the included guidelines/pathways varies widely. In turn, this implies that the fulfilment of integrated PC criteria is generally not taken into consideration when designing cancer guidelines/pathways. Previous studies looking at End-of-Life care content in medical textbooks and in treatment guidelines for life-limiting disease already revealed that top-selling textbooks and guidelines on chronic, life-limiting illnesses offer little information on caring for patients at the end of life [123]. Our findings reveal that five of the guidelines/pathways fulfilled at least 10 out of 11 IPC criteria (in fact three fulfilled all 11 criteria).

A closer look at the content of these guidelines has shown that they offer similar recommendations on IPC (Table 5). This has a three-fold importance. First it affirms that these five guidelines can serve as a benchmark for PC integration in cancer guidelines. Second, it hints that for all the guidelines/pathways that scored moderately or low, there is a considerable room for improvement. Finally, it supports the appropriateness of using Emanuel’s tool for the evaluation of the PC content of guideline data.

To conclude, it can be inferred that, even though small discrepancies do exist, the strategies proposed by the guidelines/pathways that scored the highest have strong content similarity. In turn, this suggests that there is a common conception concerning the incorporation of integrated PC into the framework for guidelines/pathways.

Finally, it is important to reiterate that we should differentiate between the completeness of guidelines/pathways content and its performance following its clinical implementation. Although a correlation between the two is quite likely to exist, a high score with respect to Emmanuel’s criteria cannot be considered to necessarily speculate on the efficacy of the guidelines/pathways in the clinical context. Future research could explore the clinical utility of these top guidelines/pathways.

### Study limitations

As is the case with any study that aspires to conduct a systematic literature review, a major limitation stems from the choice of the search strategy. We started with a broad search not only focussing on cancer but also on chronic disease. The results of the latter will be the subject of a subsequent article. More specifically, the search of the electronic databases returned a large amount of articles, many of which originated outside Europe.

In general, there is a lack of standardised definitions regarding integrated PC [8]. For the needs of this study, we furnished our own functional definition. However, our results might differ if a different definition had been employed.

An additional limitation stems from the confinement to guidelines/pathways published in Dutch, English, French, German, Hungarian and Spanish. Hence, one may not exclude the possibility that more guidelines and pathways exist in other languages spoken in the EU.

As mentioned above, the evaluation of the content of the guidelines/pathways used Emmanuel’s criteria. The authors of this study, in collaboration with all the InSup-C project partners, decided to use this tool on the basis of its apparent completeness. Therefore, one may not exclude the possibility that a different assessment tool would lead to a different score of the guidelines/pathways and thus a different hierarchy. Additionally, we only performed a limited analysis of the content of recommendations of IPC based on the most complete guidelines for PC according to Emmanuel’s criteria.

Finally, the fact that we included only guidelines that included only two or more of the 11 Emanuel’s IPC criteria might have skewed the results favouring a more positive view of integration of PC in oncology care in Europe.

## Conclusions

Five studies were found to fulfil at least 10/11 of the IPC criteria for completeness on IPC. These guidelines/pathways proposed very similar strategies for the realization of these criteria and were based on high levels of evidence. Consequently, they could serve as benchmarks of how PC can be integrated in cancer guidelines. As such, they also can provide a base to further investigate what constitutes integrated PC in cancer.

Our systematic review has revealed the importance of a holistic approach and interventions aimed at reducing suffering by deploying an integrated palliative care approach. Additionally, our results illustrate that there is disagreement on the appropriate referral criteria for IPC which remains a contentious and challenging topic in terms of the integration of PC in cancer care.

As mentioned above, the included guidelines/pathways do not embody aspects of implementation. Therefore, even though the theoretical framework of these guidelines/pathways conforms reasonably well to the state of the art in IPC, their applicability in practice needs to be further investigated.

Overall, our findings have identified both the strengths and the weaknesses of the available guidelines and pathways in Europe for patients with cancer in terms of the integration of palliative care. Consequently, we anticipate that our findings may inform physicians and policy makers as a framework in their efforts to improve the integration of PC in the care of people with cancer.

### Ethics/research governance approval

This is a systematic review of primary studies. Further ethical approval is not applicable.
